# Editorial: Biology of C-reactive protein

**DOI:** 10.3389/fimmu.2024.1445001

**Published:** 2024-06-19

**Authors:** Alok Agrawal, Yi Wu

**Affiliations:** ^1^ Department of Biomedical Sciences, James H. Quillen College of Medicine, East Tennessee State University, Johnson City, TN, United States; ^2^ MOE Key Laboratory of Environment and Genes Related to Diseases, School of Basic Medical Sciences, Xi’an Jiaotong University, Xi’an, China

**Keywords:** C-reactive protein, COVID-19, inflammation, pentraxin, PTX3

C-reactive protein (CRP) was discovered almost 100 years ago in the sera of patients suffering of pneumococcal pneumonia [ (1), reviewed in (2)]. Since then, there has been two major questions about CRP: how does CRP appear in the blood within hours of acute inflammation and what does CRP do in inflammatory states? The goal of this Research Topic was to cover the latest developments on the mechanisms of expression of the CRP gene and on the structure-function relationships of CRP.

The history of research on the biology of CRP since its discovery in 1930 till 1982 is summarized in a personal perspective article by Kushner. It was only during the first 50 years when three fundamental discoveries about CRP were made. One, the primary ligand-binding specificity of CRP was for phosphocholine-containing substances (3). Two, ligand-complexed CRP activated the classical pathway of the complement system [reviewed in (4)]. Three, CRP bound to Fc receptors even if CRP was not complexed with its ligand or even if there was no conformational change in the structure of CRP [reviewed in (5)].

CRP is an acute phase protein produced by the liver [reviewed in (6)]. The expression of the CRP gene in hepatocytes and the subsequent biosynthesis of the protein increase dramatically in response to pro-inflammatory cytokines produced during acute inflammation. The mechanism of CRP gene expression in acute phase is not fully understood. Recently, it has been reported that an enhancer located upstream of the proximal promoter of the CRP gene is critical for the acute phase expression of CRP (7). An original research article by Hernández-Banqué et al. reports the mechanism of expression of the porcine CRP gene. The authors show that the porcine and human CRP proximal promoter regions have been conserved, sharing binding sites for transcription factors. Like in the human CRP gene, there is a highly conserved putative enhancer on the porcine CRP gene. The enhancer-promoter interaction was found to be necessary for the acute phase induction of CRP expression in liver.

Since the serum level of CRP rises in inflammatory states, serum CRP is used as a non-specific biomarker for inflammation before and during the treatment of inflammatory diseases (6). In a perspective article by Mehta et al., the authors make a case for CRP to be used as a primary biomarker of inflammation and therefore disease progression and severity of Parkinson’s disease, particularly in studies examining the impact of an intervention on the signs and symptoms of the disease.

CRP has been related to COVID-19 caused by severe acute respiratory syndrome coronavirus-2 (SARS-CoV-2) [reviewed in (8)]. Three original research articles are presented here showing the usefulness of CRP as a biomarker of COVID-19. Hopkins et al. report that the levels of CRP are significantly raised and associated with disease severity in patients with severe COVID-19, suggesting that the serum CRP level is a useful biomarker for predicting disease severity. Their data also indicated that there was a low level of inflammation which lasted for at least six weeks following COVID-19. Molins et al. investigated the potential of monomeric CRP (mCRP, a dissociated subunit of pentameric CRP) in serum as a biomarker of disease severity in COVID-19 patients. They found that the patients with severe disease had higher levels of both pentameric CRP and mCRP. However, mCRP but not pentameric CRP was independently associated to disease severity, indicating the potential of serum mCRP levels as a biomarker of clinical severity in COVID-19. Paranga et al. show that the patients with severe COVID-19 have higher serum levels of CRP compared to the moderate cases. The data also identified CRP as the best discriminate between severe and non-severe forms of COVID-19.

Native CRP is composed of five identical subunits arranged in a cyclic pentameric symmetry (9). CRP has been shown to exist and function as a pattern recognition molecule in three different structural conformations: native pentameric CRP, non-native pentameric CRP (a transitional conformation where the pentamer is structurally altered) and mCRP. Olson et al. reviews the published literature on the functions of these three forms of CRP and conclude that pentameric CRP is anti-inflammatory while mCRP is pro-inflammatory. This review provides a revised understanding of the structure-function relationships of CRP as related to innate immunity and inflammation.

*In vitro*, CRP exhibits two functions: a recognition function and an effector function. The recognition function involves the binding of CRP to a ligand. The effector function of CRP involves complement activation by liganded CRP. Combined, these two functions of CRP have been shown to provide innate immunity against pneumococcal infection [reviewed in (10)]. In this Research Topic, there are six original research reports focusing on the functions of CRP in abdominal aortic aneurysms, inflammatory autoimmune arthritis, cancer, COVID-19, *Leishmania* parasite infection and nephritis. Fu et al. investigated the functions of CRP in abdominal aortic aneurysms employing a mouse model of the disease. They report that the serum CRP levels are higher in aneurysmal than that in non-aneurysmal aortas. CRP contributed to the pathogenesis of the disease since the deficiency of CRP was found to suppress aneurysmal aortic dilation and CRP did so by attenuating aneurysmal elastin destruction, macrophage accumulation and matrix metalloproteinase-2 expression. Singh et al. investigated the functions of CRP in inflammatory arthritis employing a mouse model of collagen-induced arthritis. They report that CRP lowers the serum level of IL-17, but not TNF-α, and decreases the incidence of collagen-induced arthritis in mice. Køstner et al. investigated the functions of CRP in cancer and within the tumor microenvironment in a colon cancer cohort. They show that mCRP is abundantly present within tumors from patients with high serum CRP levels. mCRP was detected exclusively within tumors. Some tumor cells were also found to colocalize with mCRP, suggesting a direct interaction or mCRP expression by the tumor itself. Liu et al. investigated the relationship between serum CRP levels and circulating megakaryocyte proportion in COVID-19 patients. They found that serum CRP levels correlated with megakaryocyte marker genes, and megakaryocytes were significantly accumulated in severe cases. The authors propose a model of how CRP regulates immune responses in COVID-19 infection. Seow et al. investigated the interactions between CRP and the parasite *Leishmania mexicana*. They show that CRP binds to short phosphoglycan repeats of proteophosphoglycans secreted by the parasite and subsequently activates the complement system. Liu et al. investigated the functions of CRP in lupus nephritis. They show that the majority of nephritis patients have autoantibodies to both C1q and mCRP. They also show that mCRP interacts with C1q and that this interaction inhibits the classical pathway of complement activation.

CRP is a member of the pentraxin family of proteins. The other major pentraxins are serum amyloid P component (SAP) and long pentraxin 3 (PTX3). PTX3 is the prototype of long pentraxins while CRP and SAP are short pentraxins [reviewed in (11)]. Both SAP and PTX3 have also been implicated in inflammatory diseases. A review article by Massimino et al. summarizes the current state of knowledge on PTX3. They review the biosynthesis and structure-function relationships of PTX3 in light of the most recent advances in its structural biology, with a focus on the interplay with complement and the emerging roles as a component of the extracellular matrix.

CRP is also a target for developing therapeutics. Rizo-Téllez et al. review recent advances in the strategies to therapeutically lower the serum CRP levels and the development of CRP antagonists specially an inhibitor that could change the conformation of CRP. The authors also discuss the therapeutic potential in mitigating the deleterious actions attributed to CRP under various pathologies, including cardiovascular, pulmonary and autoimmune diseases and cancer.

CRP has been conserved from arthropods to humans [reviewed in (12, 13)]. However, it remains unclear how CRP confers immunity to invertebrates against pathogens. Bhattacharya and Munshi review the published literature on the significance of the presence of CRP in invertebrates. They review the site of synthesis of CRP, the constitutive and induced levels of CRP in the plasma of invertebrates, and the primary structure of CRP from various invertebrate species. Since invertebrates lack an acquired immune response, the authors propose that the invertebrates are dependent on the multifunctional roles of CRP leading to evolutionary success of the invertebrate phyla.

In conclusion, this Research Topic contains perspective articles, review article and original research articles on the following aspects of CRP: mechanisms of expression of the CRP gene, use of CRP as a biomarker of inflammation, structure-function relationships of CRP, functions of the CRP homologs in inflammatory states, CRP in relation to COVID-19 and on the evolution of CRP across the animal kingdom.

## Author contributions

AA: Writing – original draft, Writing – review & editing. YW: Writing – original draft, Writing – review & editing.
